# Do shapes have feelings? Social attribution in children with autism spectrum disorder and attention-deficit/hyperactivity disorder

**DOI:** 10.1038/s41398-021-01625-y

**Published:** 2021-09-25

**Authors:** Marlee M. Vandewouw, Kristina Safar, Sarah I. Mossad, Julie Lu, Jason P. Lerch, Evdokia Anagnostou, Margot J. Taylor

**Affiliations:** 1 Department of Diagnostic Imaging, Hospital for Sick Children, Toronto, ON Canada; 2 Program in Neurosciences & Mental Health, Hospital for Sick Children, Toronto, ON Canada; 3 Autism Research Center, Bloorview Research Institute, Holland Bloorview Kids Rehabilitation Hospital, Toronto, ON Canada; 4 Institute of Biomedical Engineering, University of Toronto, Toronto, ON Canada; 5 Department of Psychology, Hospital for Sick Children, Toronto, ON Canada; 6 Wellcome Centre for Integrative Neuroimaging, FMRIB, Nuffield Department of Clinical Neurosciences, University of Oxford, Oxford, United Kingdom; 7 Department of Medical Biophysics, University of Toronto, Toronto, ON Canada; 8 Institute of Medical Science, University of Toronto, Toronto, ON Canada; 9 Department of Medical Imaging, University of Toronto, Toronto, ON Canada; 10 Department of Psychology, University of Toronto, Toronto, ON Canada

**Keywords:** ADHD, Autism spectrum disorders, Neuroscience

## Abstract

Theory of mind (ToM) deficits are common in children with neurodevelopmental disorders (NDDs), such as autism spectrum disorder (ASD) and attention-deficit/hyperactivity disorder (ADHD), which contribute to their social and cognitive difficulties. The social attribution task (SAT) involves geometrical shapes moving in patterns that depict social interactions and is known to recruit brain regions from the classic ToM network. To better understand ToM in ASD and ADHD children, we examined the neural correlates using the SAT and functional magnetic resonance imaging (fMRI) in a cohort of 200 children: ASD (*N* = 76), ADHD (*N* = 74) and typically developing (TD; *N* = 50) (4–19 years). In the scanner, participants were presented with SAT videos corresponding to social help, social threat, and random conditions. Contrasting social vs. random, the ASD compared with TD children showed atypical activation in ToM brain areas—the middle temporal and anterior cingulate gyri. In the social help vs. social threat condition, atypical activation of the bilateral middle cingulate and right supramarginal and superior temporal gyri was shared across the NDD children, with between-diagnosis differences only being observed in the right fusiform. Data-driven subgrouping identified two distinct subgroups spanning all groups that differed in both their clinical characteristics and brain–behaviour relations with ToM ability.

## Introduction

The ability to attribute mental states to oneself and to others while understanding that the mental states of others are independent of one’s own is a crucial aspect of adaptive and appropriate social interactions. This ability, called the theory of mind [1] (ToM), emerges at preschool age and continues to develop over the lifespan [2, 3]. This complex social-cognitive ability is supported by a rich network of brain regions including the temporal-parietal junction (TPJ), medial prefrontal cortices, and superior temporal gyri in both children [4, 5] and adults [6]. Children with neurodevelopmental disorders (NDDs) such as autism spectrum disorder [7] (ASD) and attention-deficit/hyperactivity disorder [8] (ADHD) commonly present with ToM deficits, which contribute to poorer development of social-cognitive skills.

Owing to increasing awareness of the high rates of overlap and comorbid symptoms in ASD and ADHD [9, 10], behavioural ToM atypicalities in children with these two disorders have begun to be studied together in comparison to typically developing (TD) children [11–14]. In behavioural studies, children with ASD performed more poorly on ToM tasks than both ADHD and TD children [11, 14], with the ADHD children showing less pronounced, but still present, difficulties compared with TD children [12, 13].

Heider et al. [15] first introduced a ToM task, called the social attribution task (SAT), which consists of animations of simple geometric shapes within an environment; the shapes move in ways that elicit automatic attribution of social interactions to the movements. Unlike many other ToM tasks, there are no complex language-dependent instructions, nor are there explicit portrayals of people and emotions, making it accessible for use with children and clinical populations. The SAT was originally a behavioural task, where the participants were asked to narrate the animations and their responses were coded on their attribution of social meaning to the sequences [16], but has since been adapted in various forms for neuroimaging studies [5, 17–27]. Compared with trials where the geometric shapes moved randomly, the social animations engaged classic ToM regions in adults, including the medial prefrontal cortices, superior temporal sulci, TPJ, amygdalae and the fusiform gyri [25–34]. Only Moriguchi et al. [23] and Ohnishi et al. [5] have used the SAT to study ToM in small samples of TD children and adolescents, and showed activation in regions similar to adults.

The SAT has also been used to investigate ToM in individuals with ASD [16, 25, 28–32]. Behaviourally, when describing the videos depicting ToM sequences, ASD children, adolescents and adults performed worse than their TD counterparts [16, 29, 30, 32]. Neuroimaging studies have included mainly adults with ASD [25, 28, 31]. Castelli et al. [28] found that ASD adults activated similar ToM regions as TDs when watching the social interactions, but to a lesser extent. Ammons et al. [31] also showed that both TD and ASD adults activated ToM regions when watching geometric shapes and human stick figures moving in a social manner; however, typical adults activated the bilateral precuneus and superior and middle temporal regions to a greater extent than ASD adults. Using other ToM protocols, such as social stories and mentalizing, fMRI studies have investigated the neural underpinnings of ToM deficits in children with ASD [33–36]. Findings have shown atypical recruitment of several brain regions involved in ToM reasoning in ASD, with patterns of both increased [33, 34] and decreased activation [35, 36] compared with TD children.

A recent fMRI study examined the neural correlates of ToM in a large TD vs. ASD study including participants from 6 to 30 years of age [37]. A similar task to the SAT (the Frith-Happé triangles) was used including three different conditions: ToM, goal-directed and random movement. In contrast to the above studies, they found no differences in social brain activity with either age or group. Interestingly, this same task was used in four groups of adults: TD, ADHD, ASD and comorbid ADHD + ASD [38]. When comparing ToM and random animations, reduced activation of key temporal-parietal ToM areas was found in ADHD compared to ASD alone and comorbid ASD + ADHD groups, indicating functional neural deficits in ADHD. Although behavioural studies in children with ADHD have shown both intact ToM [39, 40] and ToM deficits [41, 42] particularly with regards to understanding emotions [43–45], no neuroimaging studies have examined ToM in children with ADHD.

Thus, the present study is the first to examine the neural mechanisms supporting ToM during the SAT in children with ASD and ADHD, to establish whether these mechanisms differ in comparison to their TD peers. Also, importantly, given the overlap in social-cognitive difficulties in these two NDDs, we wanted to determine whether they had shared or distinct neural correlates of their challenges with ToM understanding. We hypothesised that both the ASD and ADHD children would show hypoactivation in ToM brain regions such as the TPJ, temporal and medial frontal cortices, compared with TD children, but these effects would be more marked in the ASD group.

## Materials and methods

### Participants

Participants were recruited through the Province of Ontario Neurodevelopmental Disorders (POND) network, and participants who completed the full imaging protocol between November 2016 and January 2020 were selected for this study. The cohort included children and adolescents 5–19 years of age who were either TD (*N* = 55, 40 males) or received a primary diagnosis based on expert clinical judgment of ASD (*N* = 81, 62 males), confirmed with the Autism Diagnostic Observation Schedule-2 [46] and Autism Diagnostic Interview-Revised [47], or ADHD (*N* = 88, 68 males), confirmed with the Kiddie Schedule for Affective Disorders and Schizophrenia [48] and Parent Interview for Child Symptoms [49]. TD participants with a history of prematurity, neurodevelopmental, psychiatric or neurological diagnoses, or who have a first-degree relative with an NDD were not included. Participants were not excluded on the presence of comorbidities or usage of psychotropic medication (see Supplemental Tables 1 and 2). Clinical behavioural measures were obtained (see Supplemental Information for further details).

### Social Attribution Task (SAT)

**Fig. 1:**
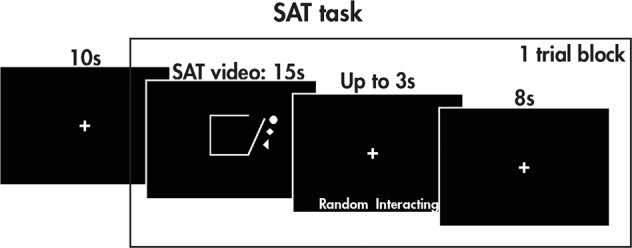
The Social Attribution Task (SAT). The SAT task consisted of trial blocks with a social or random video (15 s), a prompt asking the participant about the nature of the video (random or interacting, up to 3 s long depending on response time), and a rest period (8 s). Each run consisted of an initial ten-second rest period to acclimate the participant to the scanning environment, followed by eight trial blocks with two social help, two social threat, and four random videos presented in a randomised order; a total of three runs were collected.

The SAT, adapted by Klin et al. [16] and Schultz et al. [24] for neuroimaging, consists of 15 s videos of three shapes in motion, designed to elicit social attributions to the moving shapes or not (see Fig. 1 and Supplemental Information for further details). The videos were classified into two conditions: social and random; their order was randomised across runs. The videos in the social condition were further categorised as either social help or social threat. After the videos, ‘random’ or ‘interacting’ appeared on the screen and the children responded with a button press to indicate which word described the video. After completing the MRI session, the videos were replayed to the participants outside the scanner, and they were asked “Tell me everything the shapes are doing”. For each video description, the responses were recorded and scored based on Klin [16] to extract (a) the number of words used, (b) the number of errors (vague references, misattributions, irrelevant and inconsistent attributions) and (c) the animation index, which summarises a participant’s ability to socially attribute meaning to the video. Repeated measures ANOVAs were performed to investigate the behavioural measures (word count, errors, animation index), with condition (social, random) as the within-subject factor and diagnosis (TD, ASD, ADHD) as the between-subjects factor.

### fMRI voxelwise analyses

fMRI data were acquired during the SAT and supplemented by a T1-weighted structural image for registration (see the Supplementary Information for further acquisition and preprocessing details). After preprocessing, time-series analyses were performed on each block using FMRIB’s Improved Linear Model [50]. The task conditions (social, random, baseline) were used as explanatory variables and convolved with a hemodynamic response function, and the pairwise contrasts of the task conditions (social vs. baseline, random vs. baseline, social vs. random), were examined, controlling for signals from the white matter, cerebrospinal fluid and six motion parameters. Within each participant, contrasts were averaged across blocks using FSL’s FMRI Expert Analysis Tool with fixed effects. Group-level analyses were performed using FMRIB’s Local Analysis of Mixed Effects [51]. The across-group, within-group and between-group effects of the social vs. random and social help vs. threat contrasts were examined, covarying for age and sex. (*Z* = 2.3, *p*_corr_ < 0.05, Gaussian Random Field theory familywise error corrected). The main effects of age and diagnosis-by-age interactions were also investigated. For all analyses, significant clusters were localised using the Automated Anatomical Labelling (AAL) atlas [52]. Brain–behaviour relations were investigated between each significant cluster identified by a pairwise between-group test and the NEPSY-TM. See Supplementary Information for further details.

### Data-driven subgrouping

Given the heterogeneity within ASD and ADHD and shared symptomology across the disorders, a diagnosis-agnostic subgrouping approach was used across the TD, ASD and ADHD participants. For each of the four main effects examined (bidirectional social vs. random, social help vs. social threat), the mean subject-level COPE values were extracted across the significant voxels for each participant. The four sets of COPE values were each regressed against age and sex, and the residuals were *z* scored and used together as observations in the subgrouping analysis (see Supplementary Information).

## Results

### Participant demographics and behavioural measures

From an initial sample of 223 participants, after removing those with excessive motion, data from 50 TD, 76 ASD and 74 ADHD children remained (Table 1; see Supplementary Information for statistical details on demographics and clinical behavioural measures).

**Table 1:** Participant demographics and descriptive statistics for the clinical behavioural measures for the TD, ASD, and ADHD participants.

	TD	ASD	ADHD
*N*	50	76	74
Sex (M:F)	36:14	58:18	56:18
Mean age (years; ±std.)	12.27 ± 4.19	12.81 ± 3.49	12.23 ± 2.92
Mean FD (mm; ±std.)	0.19 ± 0.06	0.21 ± 0.07	0.20 ± 0.07
Mean FSIQ (±std.)	111 ± 11	97 ± 20	105 ± 13
Mean CBCL-AP (±std.)	55 ± 8	88 ± 9	92 ± 10
Mean SCQ-TOT (±std.)	2 ± 2	19 ± 7	6 ± 5
Mean ABAS-GAC (±std.)	100 ± 14	69 ± 14	83 ± 14
Mean NEPSY-TM (±std.)	24 ± 3	21 ± 5	24 ± 3

*TD* typically developing, *ASD* autism spectrum disorder, *ADHD* attention-deficit/hyperactivity disorder, *M* male, *F* female, *std* standard deviation, *FD* framewise displacement, *FSIQ* full-scale intelligence quotient, *CBCL-AP* Child Behaviour Checklist attention problem subscale, *SCQ-TOT* Social Communication Questionnaire total scale, *ABAS-GAC* Adaptive Behaviour Assessment System’s General Adaptive Composite score, *NEPSY-TM* Developmental Neuropsychological Assessment Theory of Mind total score.

With the behavioural measures obtained during the video descriptions outside the scanner (summarised in Table 2), participants used more words to describe the social compared with random videos (*F*(1,193) = 308.49, *p* = 8.28 × 10^−42^); there was no difference in word count amongst the three diagnostic groups (*F*(2,192) = 1.51, *p* = 0.22) nor a group-by-condition interaction (*F*(2,197) = 2.53, *p* = 0.08). The diagnostic groups differed, however, in the number of errors made (*F*(2,197) = 4.90, *p* = 0.01); post hoc tests revealed that TD made fewer errors than the ASD (*p* = 0.02) and ADHD groups (*p* = 2.75 × 10^−3^). There was no main effect of social vs. random condition (*F*(2,197) = 0.77, *p* = 0.38) nor a group-by-condition interaction (*F*(2,197) = 1.02, *p* = 0.36) on the number of errors. The animation index was higher to the social compared with random videos (*F*(1,198) = 582.64, *p* = 4.61 × 10^−60^). Although there was no main effect of diagnosis (*F*(2,197) = 1.58, *p* = 0.21), there was a group-by-condition interaction (*F*(2,197) = 3.94, *p* = 0.02): while there were no between-group differences in the animation index when describing the random videos, the ASD participants scored lower than both the TD (*p* = 0.02) and ADHD (*p* = 4.86 × 10^−3^) participants when describing the social videos. Descriptive statistics for the accuracy and reaction time to the picture question during the task are reported in Supplemental Table 3.

**Table 2:** Means and standard deviations for the task behavioural measures (word count, number of errors, and animation index) for the TD, ASD, and ADHD participants, along with statistical results from repeated-measure ANOVAs examining main effects of the condition, group, and their interaction.

		TD	ASD	ADHD
Word count	Mean social (±std.)	28 ± 13	23 ± 15	26 ± 13
	Mean random (±std.)	15 ± 9	13 ± 10	14 ± 10
	Main effect of condition	*F(1,193) = 308.49, *p* = 8.28 × 10^−42^: S > R		
	Main effect of group	*F*(2,192) = 1.51, *p* = 0.22		
	Group-by-condition interaction	*F*(2,197) = 2.53, *p* = 0.08		
# Errors	Mean social (±std.)	0.35 ± 0.36	0.52 ± 0.46	0.52 ± 0.40
	Mean random (±std.)	0.27 ± 0.28	0.47 ± 0.42	0.56 ± 0.70
	Main effect of condition	*F*(2,197) = 0.77, *p* = 0.38		
	Main effect of group	*F(2,197) = 4.90, *p* = 0.01: TD < ASD, ADHD		
	Group-by-condition interaction	*F*(2,197) = 1.02, *p* = 0.36		
Animation index	Mean social (±std.)	2.12 ± 0.41	1.92 ± 0.57	2.14 ± 0.42
	Mean random (±std.)	1.05 ± 0.42	1.06 ± 0.50	1.04 ± 0.46
	Main effect of condition	*F(1,198) = 582.64, *p* = 4.61 × 10^−60^: S > R		
	Main effect of group	*F*(2,197) = 1.58, *p* = 0.21		
	Group-by-condition interaction	*F(2,197) = 3.94, *p* = 0.02: S, ASD < TD, ADHD		

Significant results are highlighted by an asterisk. *TD* typically developing, *ASD* autism spectrum disorder, *ADHD* attention-deficit/hyperactivity disorder, *S* social, *R* random, *std* standard deviation.

### fMRI analyses

#### Main effects of the condition

**Fig. 2:**
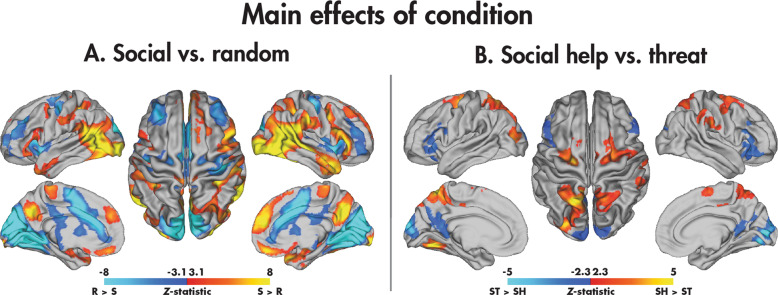
Main effects of the condition in the SAT. Significant (*p*_corr_ < 0.05) across-group main effects are shown for the social vs. random (**A**) and social help vs. social threat (**B**) contrasts.

Across the TD, ASD and ADHD groups, there was widespread differential activation of the brain between the social and random videos (Fig. 2A; Table 3). Activation was increased to the social compared with random videos in the bilateral inferior and middle occipital gyri, supramarginal gyri and temporal poles. Other regions included more extensive activation in the right hemisphere, including the right middle temporal, amygdala, supramarginal and angular gyrus, and inferior frontal regions. Activation was increased to the random compared with social videos in regions predominantly localised to the medial occipital and cingulate cortices. Contrasting the social help and threat conditions (Fig. 2B; Table 3), the social help videos activated the bilateral superior parietal and precentral gyri, precuneus, and dorsolateral superior frontal gyri and left ventromedial occipital regions more than social threat videos. In contrast, the threat condition induced increased activation in the bilateral medial occipital and inferior frontal cortices.

**Table 3:** Brain regions showing significant main effects of condition (across groups) for the social vs. random and social help vs. threat contrasts (*Z* > 2.3, *p*_corr_ < 0.05).

Contrast	Cluster	N_voxels_	*p*_corr_	Max *Z*	Max *Z* coordinates (*x*, *y*, *z*) (mm)	AAL regions
Social>random	1	37200	0	12.40	(48, −68, −6)	IOG.R, MTG.R, SMG.R, IFGo.R, ANG.R, MOG.R, HIP.R, SFGm.R, REC.L, AMYG.R, TPOm.R
	2	14173	7.26^−31^	11.40	(−26, −94, −4)	IOG.L, SMG.L, MOG.L
	3	4954	9.51e^−15^	6.73	(−22, −6, −20)	TPOm.L
Random>social	1	54149	0	14.30	(−12, −94, 22)	CAL.L, CUN.R, HES.R, LING.L, CAL.R, CUN.L, LING.R, PUT.R, SOG.L, ACG.L, MCG.R, INS.L, MCG.L, INS.R, PUT.L
Social help>social threat	1	3597	7.23e^−12^	5.50	(−18, −58, 60)	SPG.L, PCUN.L, SPG.R, MOG.L, SOG.L, PCUN.R
	2	1507	3.81e^−6^	4.09	(28, −6, 52)	SFGd.R, SMA.R, PreCG.R
	3	1169	5.20e^−5^	5.78	(−26, −68, −16)	FFG.L, LING.L
	4	1019	1.79e^−4^	4.84	(−22, −16, 58)	SFGd.L, PreCG.L
	5	532	1.67e^−2^	3.97	(60, −26, 30)	SMG.R, IPL.R
Social threat>social help	1	3421	1.94e^−11^	6.33	(14, −84, 4)	CAL.R, CUN.L, CUN.R, CAL.L, LING.R, SOG.L, PCUN.R, PCUN.L, SOG.R
	2	1153	5.91e^−5^	4.52	(42, 34, −8)	ORBi.R, IFGt.R, ORBm.R
	3	914	4.42e^−4^	3.90	(−32, 28, 0)	ORBi.L, IFGt.L, IFGo.L

*N*_voxels_ Number of voxels, *p*_corr_ corrected *p* value, *Z* *Z*-statistic, *AAL* Automated Anatomical Labelling atlas, *TD* typically developing, *ASD* autism spectrum disorder, *ADHD* attention-deficit/hyperactivity disorder, R right, *L* left, *IOG* inferior occipital gyrus, *MTG* middle temporal gyrus, *SMG* supramarginal gyrus, *IFGo* opercular part of the inferior frontal gyrus, *ANG* angular gyrus, *MOG* middle occipital gyrus, *HIP* hippocampus, *SFGm* medial superior frontal gyrus, *REC* gyrus rectus, *AMYG* amygdala, *TPOm* pole of the middle temporal gyrus, *CAL* calcarine fissure and surrounding cortex, *CUN* cuneus, *HES* Heschl’s gyrus, *LING* lingual gyrus, *PUT* putamen, *SOG* superior occipital gyrus, *ACG* anterior cingulate gyrus, *MCG* middle cingulate gyrus, *INS* insula, *SPG* superior parietal gyrus, *PCUN* precuneus, *SFGd* dorsolateral superior frontal gyrus, *SMA* supplemental motor area, *PreCG* precentral gyrus, FFG fusiform gyrus.

#### Between-group differences: social vs. random

**Fig. 3:**
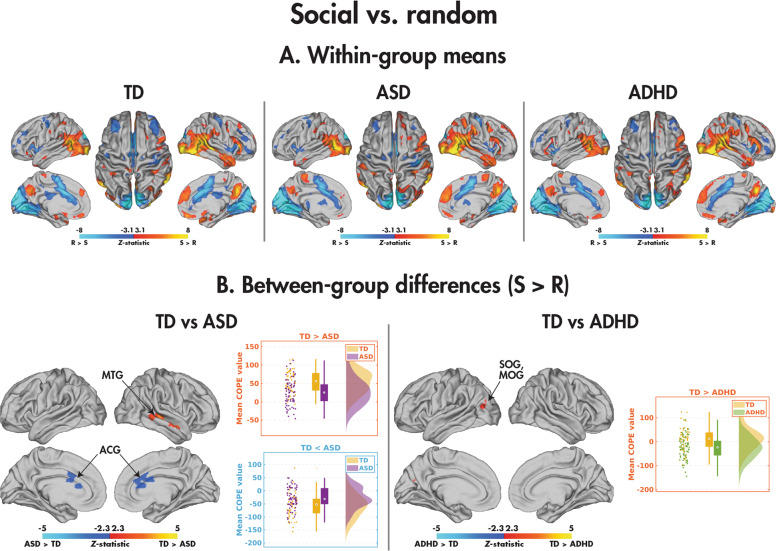
Comparing the social and random conditions in the SAT. Significant (*p*_corr_ < 0.05) within-group effects are shown for the TD, ASD, and ADHD participants (**A**), and significant (*p*_corr_ < 0.05) between-group effects are shown for the TD vs. ASD and TD vs. ADHD participants (**B**); no differences were observed between the ASD and ADHD groups.

Within the TD, ASD and ADHD groups (Fig. 3A; Table 4), significant differences between the social and random conditions appeared similar. Pairwise between-group differences were found, however, between the TD and both the ASD and ADHD participants for the social compared to random contrast (Fig. 3B; Table 4). The TD youth demonstrated significantly greater activation in the right middle temporal gyrus. The mean COPE values revealed that while both diagnostic groups recruited this region more when processing the social than random videos, the TD participants did so to a greater degree. Comparatively, the TD children showed decreased activation compared to the ASD children in the bilateral anterior cingulate gyrus (ACG). Although both groups activated this region more to the random than social videos, the effect in the TD participants was more substantial. The TD children also showed increased activation in the left superior and middle occipital gyri compared to those with ADHD; whereas the TD activated this region more to the social than random videos, the ADHD showed the reverse pattern. There were no significant group differences between the ASD and ADHD youth, nor did any of the identified clusters in the pairwise comparisons relate to the NEPSY-TM (see Supplemental Table 4). The main effects of age and diagnosis-by-age interactions are presented in Supplemental Tables 5 and 6 and Supplemental Fig. 1.

**Table 4:** Brain regions showing significant between-group differences in the social > random contrast (*Z* > 2.3, *p*_corr_ < 0.05).

Contrast	Cluster	N_voxels_	*p*_corr_	Max *Z*	Max Z Coordinates (*x*, *y*, *z*) (mm)	AAL regions
TD > ASD	1	581	0.01	4.03	(46, −28, −2)	MTG.R
TD < ASD	1	548	0.02	3.65	(14, 30, 12)	ACG.R, ACG.L
TD > ADHD	1	439	0.048	3.92	(−28, −86, 26)	SOG.L, MOG.L

*N*_*voxels*_ Number of voxels, *p*_corr_ corrected *p* value, *Z* *Z*-statistic, *AAL* Automated Anatomical Labelling atlas, *TD* typically developing, *ASD* autism spectrum disorder, *ADHD* attention-deficit/hyperactivity disorder, *R* right, *L* left, *MTG* middle temporal gyrus, *ACG* anterior cingulate gyrus, *SOG* superior occipital gyrus, *MOG* middle occipital gyrus.

#### Between-group differences: social help vs. social threat

**Fig. 4:**
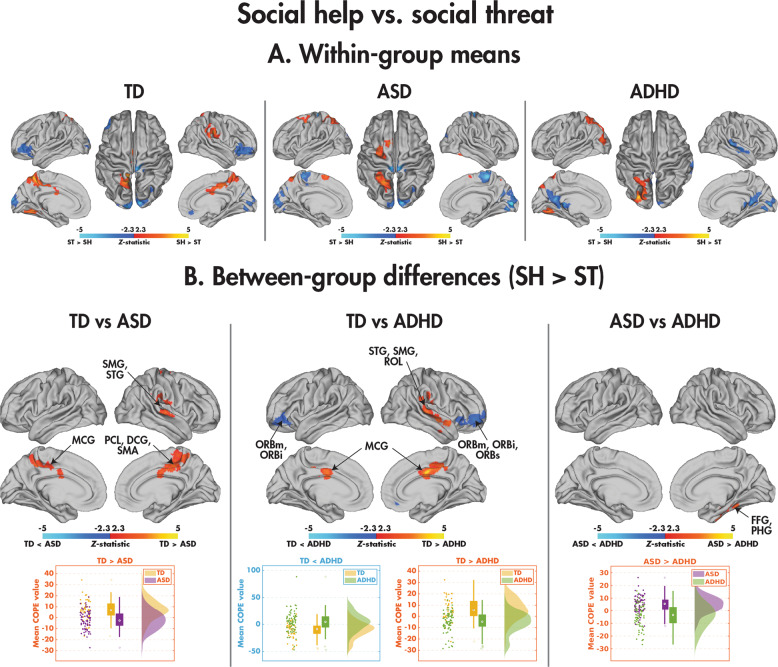
Comparing the social help and social threat conditions in the SAT. Significant (*p*_corr_ < 0.05) within-group effects are shown for the TD, ASD, and ADHD participants (**A**), and significant (*p*_corr_ < 0.05) between-group effects are shown for the TD vs. ASD, TD vs. ADHD, and ASD vs. ADHD participants (**B**).

The within-group means representing the differences between the social help and social threat conditions are presented in Fig. 4A and Table 5, with pairwise group comparisons in Fig. 4B and Table 5. Comparing the TD and ASD groups, the TD children activated the bilateral middle cingulate gyri, the right paracentral lobule, supplementary motor area, supramarginal gyrus and superior temporal gyrus more to the social help than social threat condition, whereas the ASD children showed the opposite effect, greater activation in these regions to social threat than social help. Group-by-condition interactions were also found between the TD and ADHD participants; the TD children activated the bilateral middle cingulate cortex, right supramarginal and superior temporal gyri more to the social help than social threat videos, whereas the ADHD participants showed the opposite effect. The TD participants also activated the bilateral orbital frontal cortices more to the social threat than help condition, whereas the ADHD participants showed the opposite pattern. Finally, a between-group difference was observed in the NDD children in a cluster including the right fusiform and parahippocampal gyri: the ASD recruited this region more to the social help compared with social threat videos, whereas the ADHD showed no difference. None of the identified clusters in the pairwise comparisons related to the NEPSY-TM (see Supplemental Table 4). The main effects of age and diagnosis-by-age interactions are presented in Supplemental Tables 5 and 6, and Supplemental Fig. 1.

**Table 5:** Brain regions showing significant between-group differences in the social help > social threat contrast (*Z* > 2.3, *p*_corr_ < 0.05).

Contrast	Cluster	*N*_voxels_	*p*_corr_	Max *Z*	Max Z coordinates (*x*, *y*, *z*) (mm)	AAL regions
TD > ASD	1	1603	1.91e^−6^	4.40	(8, −22, 42)	PCL.R, MCG.R, MCG.L, SMA.R
	2	533	0.02	3.69	(70, −24, 0)	SMG.R, STG.R
TD > ADHD	1	1009	1.95e^−4^	4.57	(2, −4, 34)	MCG.R, MCG.L
	2	963	2.89e^−4^	4.40	(58, −10, −8)	STG.R, SMG.R, ROL.R
TD < ADHD	1	1057	1.30e^−4^	4.14	(38, 54, −8)	ORBm.R, ORBs.R, ORBi.R
	2	568	0.01	4.04	(−36, 40, −12)	ORBm.L, ORBi.L
ASD > ADHD	1	985	2.39e^−4^	3.95	(24, −54, −34)	PHG.R, FFG.R

*N*_*voxels*_ Number of voxels, *p*_corr_ corrected *p* value, *Z* *Z*-statistic, *AAL* Automated Anatomical Labelling atlas, *TD* typically developing, *ASD* autism spectrum disorder, *ADHD* attention-deficit/hyperactivity disorder, *R* right, *L* left, *PCL* paracentral lobule, *MCG* middle cingulate gyrus, *SMA* supplemental motor area, *SMG* supramarginal gyrus, *STG* superior temporal gyrus, *ROL* rolandic operculum, *ORBm* orbital part of the middle frontal gyrus, *ORBs* orbital part of the superior frontal gyrus, *ORBi* orbital part of the inferior frontal gyrus, *PHG* parahippocampal gyrus, *FFG* fusiform gyrus.

#### Data-driven subgrouping

**Fig. 5:**
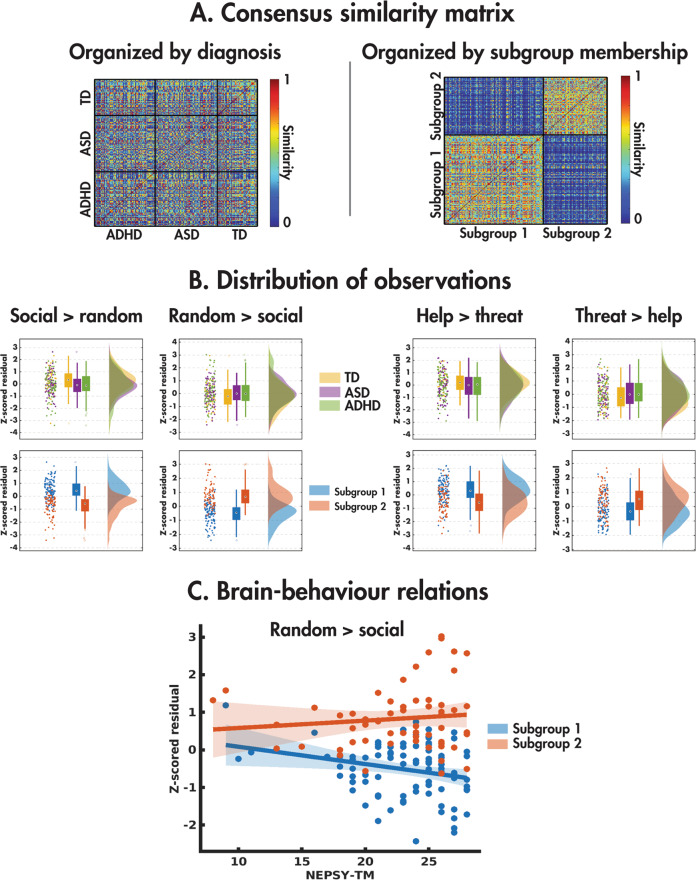
Results of the data-driven subgrouping analysis. The consensus similarity matrix organised by the diagnostic group is shown (**A**) alongside its reorganisation by cluster membership, with the distributions (**B**) of the four observations used in the clustering in the diagnostic groups (top) and subgroups (bottom). A significant brain–behaviour interaction was found with the NEPSY-TM (**C**).

The consensus similarity matrix was constructed from the *z* scored age and sex-regressed residuals of the mean COPE values across the four main effects of the condition (pairwise social vs. random and social help vs. social threat). The eigengap heuristic revealed that the optimal number of subgroups was two. The consensus similarity matrix organised by the diagnostic group is shown in Fig. 5A alongside its reorganisation by subgroup membership, highlighting the lack of similarity within the diagnostic groups. Figure 5B shows the distribution of the four observations used in the subgrouping in the diagnostic groups (top) and subgroups (bottom). Although the diagnostic groups did not differ in any of the observations, the subgroups differed on all four observations (social > random: *H*(2) = 91.83, *p* = 9.44e^−22^; random>social: *H*(2) = 98.76, *p* = 2.85e^−23^; social help>threat: *H*(2)=36.71, *p* = 1.37e^−9^; social threat>help: *H*(2) = 27.16, *p* = 1.87e^−7^). Examining the subgroup means, subgroup 1 was characterised by increased activity to the social compared with random and social help compared with threat, with subgroup 2 showing the opposite pattern (Fig. 5C).

The demographic information and descriptive statistics for the clinical behavioural measures for the data-driven subgroups are presented in Table 6. The subgroups did not differ in age (*H*(2);= 0.10, *p* = 0.75), mean FD (*H*(2) = 0.76, *p* = 0.38) or sex ratio (*χ*^2^(2, *N* = 200) = 0.17, *p* = 0.67). A chi-squared test across all subgroups revealed that the proportion of ASD, ADHD and TD participants did not differ among the subgroups (*χ*^2^(2, *N* = 200) = 5.11, *p* = 0.08), although this was trending towards significance, the majority of TDs belonging to subgroup 1. For clinical measures, those in subgroup 1 were overall better performing. Compared with subgroup 2, participants in subgroup 1 had a higher FSIQ (*H*(2) = 4.41, *p* = 0.04), and scored lower on the CBCL-AP (*H*(2) = 6.45, *p* = 0.01). The subgroup did not differ on SCQ-TOT (*H*(2) = 1.95, *p* = 0.16), ABAS-GAC (*H*(2) = 1.19, *p* = 0.27), or NEPSY-TM (*H*(2) = 0.06, *p* = 0.80).

**Table 6:** Participant demographics and descriptive statistics for the clinical behavioural measures for the data-driven subgroups.

	Subgroup 1	Subgroup 2
*N*	121	79
(*N*_ASD_, *N*_ADHD_, *N*_TD_)	(41, 43, 37)	(33, 33, 13)
Sex (M:F)	92:29	58:21
Mean age (years; ±std.)	12.06 ± 3.48	12.51 ± 3.50
Mean FD (mm; ±std.)	0.20 ± 0.07	0.20 ± 0.07
Mean FSIQ (±std.)	106 ± 17	100 ± 16
Mean CBCL-AP (±std.)	79 ± 19	86 ± 16
Mean SCQ-TOT (±std.)	10 ± 9	12 ± 10
Mean ABAS-GAC (±std.)	83 ± 18	80 ± 19
Mean NEPSY-TM (±std.)	23 ± 4	23 ± 5

*TD* typically developing, *ASD* autism spectrum disorder, *ADHD* attention-deficit/hyperactivity disorder, *M* male, *F* female, *std* standard deviation, *FD* framewise displacement, *FSIQ* full-scale intelligence quotient, *CBCL-AP* Child Behaviour Checklist attention problem subscale, *SCQ-TOT* Social Communication Questionnaire total scale, *ABAS-GAC* Adaptive Behaviour Assessment System’s General Adaptive Composite score, *NEPSY-TM* Developmental Neuropsychological Assessment Theory of Mind total score.

Finally, we performed the brain–behaviour ANCOVAs on the four observations, with subgroup membership as the factor and NEPSY-TM as the covariate (Supplemental Table 7, Fig. 5C). Subgroup 1 showed a negative relation (*F*(1,144) = 4.80, *p* = 0.03) between the NEPSY-TM and the *Z* scored residual from the random> social contrast (*R* = −0.25, *p* = 0.02), whereas subgroup 2 showed no relation (*R* = 0.11, *p* = 0.42).

## Discussion

This is the first investigation of the neural mechanisms underpinning ToM in the SAT in youth with ASD and ADHD compared with their TD peers. Behaviourally, the TD children showed greater proficiency in describing social and random videos compared with those with NDDs, and the TD and NDD children also showed differences in neural activation in social brain areas while watching the social videos. The only effect seen between the ASD and ADHD group was enhanced activity in the ASD compared with ADHD individuals in the right fusiform gyrus to social help vs. threat animations. We discuss these findings in detail below.

Behaviourally, there was no word count differences across the groups, but the TD children made fewer errors when describing the social and random animations than the NDD children. The animation index, the ability to attribute intentions to the interacting shapes, was significantly lower in those with ASD for the social but not random animations compared to the ADHD and TD children. Thus, although children with ASD and ADHD both demonstrated ToM deficits, they were less marked in ADHD. These findings buttress reports of poor social attribution ability in children and adolescents with ASD [16, 30, 32], and poorer performance in those with ASD compared to ADHD on social tasks [12, 13, 53].

In the neuroimaging analyses, all three groups showed greater activation to the social than random videos in classic social brain regions. However, differences emerged in between-group analyses: when contrasting social and random videos, the TD children activated the middle temporal gyrus to a greater extent to the social videos than the ASD group, whereas the ASD children showed greater activity to random than social movement. The middle temporal gyrus is critical in processing social signals and is consistently activated in ToM tasks (see [54] for a review), including social attribution [21, 31]. Our results extend those of Ammons and colleagues [31] to children, as they found that TD, but not ASD adults recruited middle temporal regions during an SAT more to social than random movements; others have also reported right middle temporal atypicalities in ASD related to ToM processes [55, 56]. Assaf and colleagues [55] also observed that children and young adults with ASD engaged the middle temporal gyrus less compared to typical controls during mentalizing and thinking about the intentions of others, supporting an impaired ability to establish mental representations of others (i.e., mind blindness) in ASD. These data taken together with the present findings, suggest that those with ASD are not processing social movements, a critical factor in understanding social behaviours and emotions, normally, likely contributing to the deficits seen in these domains in those with ASD.

Both the TD and ASD children demonstrated greater recruitment of the bilateral ACG to random than social videos, with greater differential activation in the TD than ASD participants. The ACG has a central role in integrating social information among key regions of the social brain network [57]. In addition, it is critical for attending to and tracking “other-oriented information”, which is crucial for understanding interpersonal perspectives [57]. In particular, the ACG is engaged when detecting errors in predictive coding (i.e., the discrepancy between expected vs. actual outcomes) of others’ behaviour [57]. The anterior cingulate is also vital for processing information with a high degree of ambiguity or uncertainty [58]. In the current study, it is possible that viewing the random shape animations evoked higher-level predictive errors associated with increased activity in the anterior cingulate gyri in TD children owing to greater uncertainty when deciding whether the shapes were interacting socially or randomly. Of note, the random videos in the present study were not mechanical as in other papers [22], still requiring some mentalizing to determine whether the shapes were interacting socially. Thus, reduced activation of the bilateral ACG to random videos in children with ASD suggests less engagement of this region when making prediction errors and social decision-making relative to their typical peers. Atypical processing of social prediction errors when tracking expectations of others has also been reported in ASD, associated with atypical ACG activity [59]. For example, Balsters and colleagues [59] observed an absence of ACG activation during social prediction errors in ASD, and ACG activity was shown to be associated with the severity of social deficits, such that more typical ACG activation was correlated with reduced impairment in the ASD group. The authors suggested that engagement of the ACG during social prediction errors is a critical aspect of social function in typical development, and the atypical recruitment of this region that we see in the current data would also underpin social impairments in ASD.

Exciting, novel results were also found when comparing social help and social threat animations, as this analysis is rarely completed. The TD children activated the right supramarginal and superior temporal gyri, ToM regions [5, 19, 26, 28, 60, 61], along with the bilateral middle anterior cingulate, implicated in empathy [62], more to the social help videos, whereas children with NDDs showed greater activation of these regions to social threat. Few studies have specifically examined how the nature of social interactions influences the neural mechanisms underpinning social attribution. In typical adults, differences in activity in the right posterior superior temporal sulcus have been reported when shapes engage in competitive vs. cooperative behaviour [21, 60, 61] and pro-social behaviour [63], suggesting that this region may be sensitive to the meaning and content of social interactions. We established that in addition to the right superior temporal area, TD children also preferentially engaged other social-cognitive areas when processing animations depicting social help compared with threat, compared to those with NDDs. Although previous work has shown dysfunction of these brain areas in adults with ASD and ADHD during ToM processing [38], we are the first to show that neural activation to different types of social interactions (i.e., help or threat) differs from that of typical controls. All three groups activated social brain regions in the comparison of social vs. random videos, although group differences emerged, as discussed above. The fact that further differences were found, however, between social help and threat indicates that those with NDDs are less able to distinguish these social behaviours, which would contribute to greater social difficulties for them. This also may be an important difference to target in behavioural interventions in the future.

Furthermore, in the opposite contrast of social threat vs. social help, we found that the TD children recruited bilateral orbitofrontal areas more than ADHD participants. The orbitofrontal cortex (OFC) is part of social brain circuitry important for decoding others’ emotional states and is implicated in ToM reasoning [64]. The OFC has also been shown in adults to be involved in processing facial and body expressions of anger, attributing negative emotions to others, and recognising socially inappropriate conduct [65–67]; our results in the TD group extend this to a younger age range. Although research examining the neural mechanisms underlying ToM in ADHD is scarce, it is proposed that atypical OFC function may contribute to deficits in social cognition [68, 69]. Therefore, our findings of greater activation in the OFC to social threat than help in TD children strengthens both findings delineating the typical function of this cortical area and abnormal OFC activation in children with ADHD.

Interestingly, the only significant difference between the ASD and ADHD groups was seen in the right fusiform gyrus, in the social threat vs. social help contrast. Several social attribution studies reported enhanced activity in the fusiform gyrus to social compared with random shape interactions in typical development [19, 22, 24, 26], in ASD and ADHD combined [38], as well as reduced activation in ASD compared to controls [28]. Individuals with ASD recruit this region less when interpreting the social animations and relating them to real-life interactions [24], relative to individuals with ADHD and TD [28], consistent with behavioural findings of greater social-cognitive deficits in ASD relative to ADHD [69]. Although there were clusters that emerged as significant in the TD contrasts with one NDD group that were not present in the other NDD group, in all cases, the omitted group had a mean COPE value that fell in between the other two groups (see Supplemental Fig. 2 and Supplemental Table 8).

Thus, the overlap between TD, ASD, and ADHD suggests a continuum from typical to atypical ToM neural processing, whose order critically depends on the brain region. This is also demonstrated by the subgrouping results, where diagnosis-agnostic analyses showed no group differentiation of the NDDs. The two subgroups were distinguished by behavioural metrics that crossed diagnostic boundaries, with most TDs belonging to the subgroup with higher intelligence and attentional skills. The two subgroups were also characterised by differing brain–behaviour relations with ToM ability. This strongly supports taking a trans-diagnostic approach to studying these populations and considering the NDDs and TDs to be part of the same continuum.

The fact that both NDD groups showed activation of the classic ToM brain areas to the social videos even in childhood, is encouraging for interventions aimed at improving and strengthening these social-cognitive abilities. Interventions that reinforce these networks may help reduce the difficulties these groups experience. Furthermore, therapies could pay special attention to social help vs. threat learning, as more subtle meaning of social interactions is evidently missed by ASD and ADHD youth, reflected in their poorer behavioural explanations, as well as atypical activation patterns compared to the TD youth.

In conclusion, the present study is the first to investigate the neural mechanisms underlying ToM during the SAT in youth with ASD and ADHD, and their TD peers. We established that TD children and adolescents demonstrate better behavioural abilities to attribute social meaning to the social animations compared with those with NDDs. Neuroimaging results showed that all three groups engaged classic ToM brain areas during the social compared with random scenarios; however, atypical activation of these areas in ASD and ADHD was seen when contrasting social and random videos and critically, also depended on the nature of social attributions.

## Supplementary information

Supplementary information
