# Supplementary material for: Do shapes have feelings? Social attribution in children with autism spectrum disorder and attention-deficit/hyperactivity disorder
Source: Transl Psychiatry. 2021 Sep 25;11:493. doi: 10.1038/s41398-021-01625-y (PMC8464598; doi:10.1038/s41398-021-01625-y)
Supplement: Supplementary file 1 — Supplementary information [file 41398_2021_1625_MOESM1_ESM.docx]

**Do shapes have feelings? Social attribution in children with autism spectrum disorder and attention-deficit/hyperactivity disorder**

Marlee M. Vandewouw, Kristina Safar, Sarah I. Mossad, Julie Lu, Jason P. Lerch, Evdokia Anagnostou, Margot J. Taylor

**Supplementary information**

**Social Attribution Task (SAT)**

The SAT, adapted by Klin et al.^1^ and Schultz et al.^2^ for neuroimaging, consists of 15s videos of three shapes in motion, designed to elicit social attributions to the moving shapes or not. In each video, a white circle, square and triangle were present on a black background, with a white square frame centred in the middle, with one edge hinged. During the videos, the shapes moved against the background, and the hinged square frame occasionally opened and shut (see **Figure 1**). The videos were classified into two conditions: social and random; their order was randomized across runs. In the social condition, the shapes moved in such a way to portray a social interaction over the course of the 15s. The videos in the social condition were further categorized as either social help (movement depicted positive social interaction such as cooperating, play, etc.) or social threat (movement depicted negative social interaction such as fighting, conspiring, etc. where one or two shapes were working against the other(s)). In the random condition, the shapes moved randomly across the screen, portraying no social interaction. After the videos, ‘random’ or ‘interacting’ appeared on the screen and the children responded with a left or right button press to indicate which word described the video.

**Participant demographics & behavioural measures**

Approval for this study was approved by the Hospital for Sick Children’s research ethics board, and written informed consent or assent was obtained from the primary caregiver or participant where appropriate. The presence of co-morbidities and use of psychotropic medication were noted, but participants were not excluded on this basis. Psychiatric comorbidities were assessed using the Diagnostic and Statistical Manual of Mental Disorders IV^3^ or V^4^ on 156 of the 200 participants included in the final analyses while psychotropic medications were recorded for 183 of the 200 participants included in the final analyses; further information can be found in **Supplemental Tables 1** and **2**, respectively.

**Supplemental Table 1**: Details on documented psychiatric comorbidities on the final analyzed sample.

| **Variable** | **TD** | **ASD** | **ADHD** |
| --- | --- | --- | --- |
| N | 50 | 34 | 72 |
| Number of participants with a psychiatric comorbidity | 0 | 23 | 38 |
| Comorbidity  breakdown | - | 4 ANX  3 ADHD  2 ID  2 LD  2 ADHD + ANX  1 ADHD + DB  1 OCD + ANX  1 ADHD + ID  1 ADHD + LD  1 ANX + LD  1 ID + LD  1 ADHD + ANX + DB  1 ADHD + ANX + ID  1 ADHD + ANX + LD  1 ADHD + OCD + ANX + LD | 11 LD  6 DB  4 ANX  1 ASD  6 ANX + LD  3 DB + LD  2 OCD + LD  2 ANX + DB  1 OCD + DB  2 ANX + DB + LD |

TD: typical developing; ASD: autism spectrum disorder; ADHD: attention-deficit/hyperactivity disorder, ANX: anxiety disorder; LD: learning disorder; ID: intellectual disability; DB: disruptive behaviour disorder.

**Supplemental Table 2**: Details on documented psychotropic medications on the final analyzed sample.

| **Variable** | **TD** | **ASD** | **ADHD** |
| --- | --- | --- | --- |
| N | 50 | 70 | 63 |
| Number of participants on psychotropic medication | 0 | 28 | 32 |
| Psychotropic medication breakdown | - | 10 Stimulant  5 SSRI  1 SNRI  1 AAP  1 Clonidine  1 Benzothiazole  1 Stimulant + melatonin  1 Stimulant + SSRI  1 Stimulant + Clonidine  1 Stimulant + AAP  1 SSRI + Guanfacine  1 Aminoketone + beta blocker  1 AAP + Guanfacine  1 Stimulant + AAP + Clonidine  1 AAP + anticonvulsant + Guanfacine + SSRI | 20 Stimulant  2 SNRI  2 Stimulant + AAP  2 Stimulant + Guanfacine  2 Stimulant + melatonin  1 Stimulant + SSRI  1 SNRI + SSRI  1 AAP + amino acid + SNRI  1 Stimulant + SSRI + Clonidine + melatonin |

N: sample size; AAP: atypical antipsychotic; SSRI: selective serotonin reuptake inhibitor; SNRI: selective norepinephrine reuptake inhibitor

Full-scale IQ (FSIQ) was obtained using age-appropriate Wechsler scales of intelligence^5–8^. The Child Behaviour Checklist^9^ attention problem subscale (CBCL-AP) was also administered to capture a measure of inattention characteristic of ADHD, yet also expressed in those with ASD. The Social Communication Questionnaire^10^ total score (SCQ-TOT) was used to capture of a measure of social communication problems characteristic of ASD, yet also present in those with ADHD. The Adaptive Behaviour Assessment System’s^11^ General Adaptive Composite score (ABAS-GAC) was used to capture a measure of the general adaptive function deficits found in both neurodevelopmental disorders. Finally, the Developmental Neuropsychological Assessment^12^ (NEPSY) test battery was obtained, from which we extracted the Theory of Mind total score (NEPSY-TM).

According to Shapiro-Wilks tests, age(*W*=0.98, *p*=0.01), mean FD (*W*=0.98, *p*=0.02), FSIQ (*W*=0.96, *p*=8.4×10^-4^), CBCL-AP (*W*=0.78, *p*=9.99×10^-16^), SCQ-TOT (*W*=0.89, *p*=2.05×10^-10^), ABAS-GAC (*W*=0.98, *p*=3.10×10^-3^) and NEPSY-TM (*W*=0.89, *p*=2.97×10^-8^) were non-normally distributed, and thus Kruskal-Wallis tests were used to test for differences amongst the groups. There were no significant differences in age (*H*(2)=1.25, *p*=0.54), mean FD (*H*(2)=2.36, *p*=0.31), or sex ratio (chi-squared test: *χ*^2^(2, N=200)=0.23, *p*=0.89) across the three groups. There was a significant effect of group on FSIQ (*H*(2)=21.16, *p*=2.54×10^-5^), with the ASD children having significantly lower FSIQ than the ADHD (*p*=0.03) and TD (*p*=1.52×10^-5^). There was also a main effect of group on CBCL-AP (*H*(2)=93.74, *p*=4.40×10^-21^), with the TD children scoring lower (meaning fewer difficulties) than the ADHD (*p*=9.56×10^-10^) and ASD (*p*=9.56×10^-10^) children. A significant effect of group on SCQ-TOT (*H*(2)=123.16, *p*=1.80×10^-27^) was also present, with all pairwise post-hoc comparisons significant: the TD children scored lower (fewer difficulties) than the ADHD (*p*=9.56×10^-10^) and ASD (*p*=0.01) children, and the ADHD lower than the ASD children (*p*=9.56×10^-10^). The groups differed on ABAS-GAC (*H*(2)=79.64, *p*=5.08×10^-18^), again with all post-hoc comparisons significant: the TD children performed better (higher adaptive scores) than the ADHD (*p*=4.12×10^-10^) and ASD (*p*=9.56×10^-^ ^10^) children, and the ADHD performed better than the ASD children (*p*=3.90×10^-6^). Finally, there was also a significant difference amongst the groups in NEPSY-TM (*H*(2)=17.51, *p*=1.57×10^-4^), with both TD (*p*=1.10×10^-3^) and ADHD (*p*=8.35×10^-4^) performing better than ASD participants.

Descriptive statistics of the accuracy and reaction times to the picture question during the fMRI task are presented in **Supplemental Table 3**. Repeated-measure ANOVAs revealed that neither accuracy or reaction time differed between the social and random videos (accuracy: *F*(1,198)=1.49, *p*=0.22; reaction time: *F*(1, 198)=0.65, *p*=0.42) or amongst the three diagnostic groups (accuracy: *F*(2,197)=2.57, *p*=0.08; reaction time: *F*(2,197)=0.97, *p*=0.38), nor was there a group-by-condition interaction (accuracy: *F*(2,197)=0.09, *p*=0.91; reaction time: *F*(2,197)=1.99, *p*=0.14).

**Supplemental Table 3**: Descriptive statistics for the accuracy and reaction time to the picture question during the fMRI task.

|  |  | **TD** | **ASD** | **ADHD** |
| --- | --- | --- | --- | --- |
| **Mean accuracy (%; ± std.)** | **S** | 98.15 ± 0.05 | 93.19 ± 0.16 | 91.81 ± 0.19 |
|  | **R** | 99.31 ± 0.02 | 94.03 ± 0.14 | 93.61 ± 0.17 |
| **Mean reaction time (s; ± std.)** | **S** | 0.93 ± 0.35 | 1.00 ± 0.32 | 1.02 ± 0.33 |
|  | **R** | 0.99 ± 0.38 | 0.95 ± 0.29 | 1.06 0.35 |

TD: typically developing, ASD: autism spectrum disorder, ADHD: attention-deficit/hyperactivity disorder; S: social; R: random

**Image acquisition**

Data were acquired using a Siemens 3.0T PrismaFIT MRI scanner with a 20 channel head and neck coil. Structural images were acquired using a T1-weighted 3D magnetization-prepared rapid acquisition with gradient echo (MPRAGE) sequence (TR/TE/TI: 1870/3.14/945ms; FA: 9˚; FOV: 240x256mm; number of slices: 192; resolution: 0.8mm isotropic; scan time: 5:01min), and functional images were acquired using a single-shot echo planar imaging (EPI) sequence (TR/TE: 1500/30ms; FA: 70˚; FOV: 222x222mm; number of slices: 50; resolution: 3mm isotropic) while the children performed the task. The task was displayed on MR-compatible goggles, and participants responded using an MR-compatible keypad.

**Preprocessing**

T1-weighted structural images were skull-stripped using FMRIB Software Library’s (FSL) Brain Extraction Tool^13^, and segmentations of the white matter and cerebrospinal fluid were obtained using FMRIB’s Automated Segmentation Tool^14^ and eroded for nuisance regression purposes. The T1-weighted images were subsequently used to register the functional data and segmentation masks to standard space. Functional data were slice-time and motion corrected using Analysis of Functional Neuroimages (AFNI) software^15^, extracting the six motion parameters. The motion parameters were used to calculate framewise displacement (FD), and volumes with FD>0.9mm^16^ were censored from the data. Task blocks with more than 1/3 of the volumes censored were not considered, and participants with only one block remaining were excluded from all subsequent analyses. Data were smoothed with a 6mm FWHM Gaussian kernel^17^, intensity normalized^18^, cleaned of nuisance signals by regressing contributions from the white matter, cerebrospinal fluid, whole-brain, and six motion parameters, along with their derivatives and quadratic terms^15^, and bandpass filtered between 0.01-0.2Hz^15^. FSL’s Independent Component Analysis-based Xnoiseifier^19,20^ was used to further clean the data of noise.

**fMRI voxelwise analysis**

For each statistical comparison (across-group, within-group, and between-group effects of condition), cortical and subcortical AAL regions with more than 5% of their volume overlapping with a significant cluster were reported. For the main effects of condition, only the 15 regions with the most overlap for were reported due to the large spatial extent of the significant activation. For the between-group differences, the mean subject-level contrast of parameter estimate (COPE) values across significant voxels were extracted for each participant and plotted.

For each cluster, the mean subject-level COPE value over the significant voxels was extracted for each participant in the two examined groups, and an analysis of covariance (ANCOVA) was performed with diagnosis as the factor and NEPSY-TM as the covariate. Upon finding significant (*p*<0.05) main effects of behaviour or group-by-behaviour interactions, post-hoc Pearson correlations were performed to determine directionality of effects.

**Data driven subgrouping**

The subgrouping analysis, implemented in MATLAB^21^, consisted of *k*-level runs, where *k* is the number of subgroups to be generated, and each run consisted of 50,000 bootstrap iterations. For each bootstrap iteration, a subsample of 63.2%^22^ of the *N* participants was selected (with replacement). The Euclidean distance between each pair of observations in the subsample was computed and used to partition the subsample into subgroups with the *k*-medoids clustering algorithm^23^, with the number of subgroups set to *k*. After the bootstrap iterations, an *N*×*N* similarity matrix was computed, where the (*i*th, *j*th) entry was the number of times participant *i* and participant *j* were partitioned into the same subgroup, expressed as a fraction of the number of times they were selected in the same bootstrap subsample. This procedure was run for the number of subgroups *k* ranging from 2 to 15. After the *k*-level runs, a final consensus similarity matrix was obtained by averaging over each *k*-level similarity matrix. The similarity matrix was partitioned using spectral clustering, where the optimal number of final subgroups was identified using the eigengap heuristic^24^.

Differences between the subgroups in the four observations, age, mean framewise-displacement (FD) and the clinical behavioural measures were investigated using Kruskal-Wallis tests (due to non-normal distributions) and a chi-squared test was used to test for differences in sex and diagnosis; significance was held at *p*<0.05. Brain-behaviour relations were investigated between each of the four main effect observations and the NEPSY-TM using an ANCOVA (*p*<0.05) with post-hoc Pearson correlations.

**Brain-behaviour relations with diagnosis**

Brain-behaviour relations were investigated between each significant cluster identified by a pairwise between-group test and the NEPSY-TM; results are presented in **Supplemental Table 4**.

**Supplemental Table 4:** Statistical results for the brain-behaviour relations ANCOVAs between the NEPSY-TM and each significant cluster identified in the pairwise between-group analyses.

|  | **Contrast** | **Cluster** | **AAL regions** | **Behaviour**  **(*F*, *p*-value)** | **Dx-Behaviour interaction**  **(*F*, *p*-value)** |
| --- | --- | --- | --- | --- | --- |
| Social  >  Random | TD > ASD | 1 | MTG.R | (0.23, 0.63) | (0.03, 0.87) |
|  | TD < ASD | 1 | ACG.R, ACG.L | (0.64, 0.42) | (0.14, 0.71) |
|  | TD > ADHD | 1 | SOG.L, MOG.L | (2.14, 0.14) | (0.64, 0.43) |
| Social  help  >  Social  threat | TD > ASD | 1 | PCL.R, MCG.R, MCG.L, SMA.R | (0.02, 0.90) | (0.46, 0.50) |
|  |  | 2 | SMG.R, STG.R | (0.00, 1.00) | (1.44, 0.23) |
|  | TD > ADHD | 1 | MCG.R, MCG.L | (0.50, 0.48) | (0.14, 0. 71) |
|  |  | 2 | STG.R, SMG.R, ROL.R | (0.03, 0.87) | (0.11, 0.74) |
|  | TD < ADHD | 1 | ORBm.R, ORBs.R, ORBi.R | (0.12, 0.73) | (0.62, 0.43) |
|  |  | 2 | ORBm.L, ORBi.L | (0.07, 0.79) | (0.09, 0.77) |
|  | ASD > ADHD | 1 | PHG.R, FFG.R | (1.58, 0.21) | (1.19, 0.28) |

AAL: Automated Anatomical Labeling atlas, F: *F*-statistic, Dx: diagnosis, R: right, L: left, PCL: paracentral lobule, MCG: middle cingulate gyrus, SMA: supplemental motor area, SMG: supramarginal gyrus, STG: superior temporal gyrus, ROL: rolandic operculum, ORBm: orbital part of the middle frontal gyrus, ORBs: orbital part of the superior frontal gyrus, ORBi: orbital part of the inferior frontal gyrus, PHG: parahippocampal gyrus, FFG: fusiform gyrus

**Effects of age**

Main effects of age across the diagnostic groups (ASD, ADHD, and TD) are presented in **Supplemental Table 5** and **Supplemental Figure 1A.** In the social greater than random contrast, brain activation was found to increase with age in the left angular, supramarginal and orbital part of the inferior frontal gyri. In contrast, age-related decreases were observed in the bilateral median cingulate gyri and paracentral lobules, the left precuneus and right middle frontal gyrus. For the social help greater than threat contrast, age-related increases in brain activation were observed in right temporal regions, while decreases were observed in bilateral occipital regions.

**Supplemental Table 5:** Brain regions showing significant main effects of age (across groups – ASD, ADHD, and TD) for the social greater than random and social help greater than threat contrasts (*Z* > 2.3, *p*_corr_ < 0.05).

| **Contrast** | **Cluster** | **N_voxels_** | ***p*_corr_** | **Max**  ***Z*** | **Max Z coordinates**  **(x, y, z) (mm)** | **AAL regions** |
| --- | --- | --- | --- | --- | --- | --- |
| Social  >  Random,  ↑Age | 1 | 682 | 4.16e^-3^ | 3.92 | (-52, -72, 24) | ANG.L, SMG.L |
|  | 2 | 493 | 0.03 | 3.89 | (-48, 32, -16) | ORBi.L |
| Social  >  Random,  ↓Age | 1 | 2098 | 5.96e^-8^ | 5.10 | (-12, -32, 44) | MCG.R, MCG.L, PCL.R, PCUN.L, PCL.L |
|  | 2 | 440 | 0.048 | 3.46 | (30, 40, 20) | MFG.R |
| Social help  >  Social threat  ↑Age | 1 | 492 | 0.03 | 3.97 | (64, -16, -10) | STG.R, MTG.R |
| Social help  >  Social threat  ↓Age | 1 | 1243 | 2.88e^-5^ | 3.98 | (-40, -82, -16) | IOG.L, LING.L |
|  | 2 | 559 | 0.01 | 3.65 | (18, -82, 4) | IOG.R, LING.R, CAL.R |

N_voxels_: Number of voxels, *p*_corr_: corrected *p*-value, *Z*: *Z*-statistic, AAL: Automated Anatomical Labeling atlas, ANG: angular gyrus, SMG: supramarginal gyrus, ORBI: orbital part of the inferior frontal gyrus, MCG: middle cingulate gyrus, PCL: paracentral lobule, PCUN: precuneus, MFG: middle frontal gyrus, STG: superior temporal gyrus, MTG: middle temporal gyrus, IOG: inferior occipital gyrus, LING: lingual gyrus, CAL: calcarine fissure and surrounding tissue


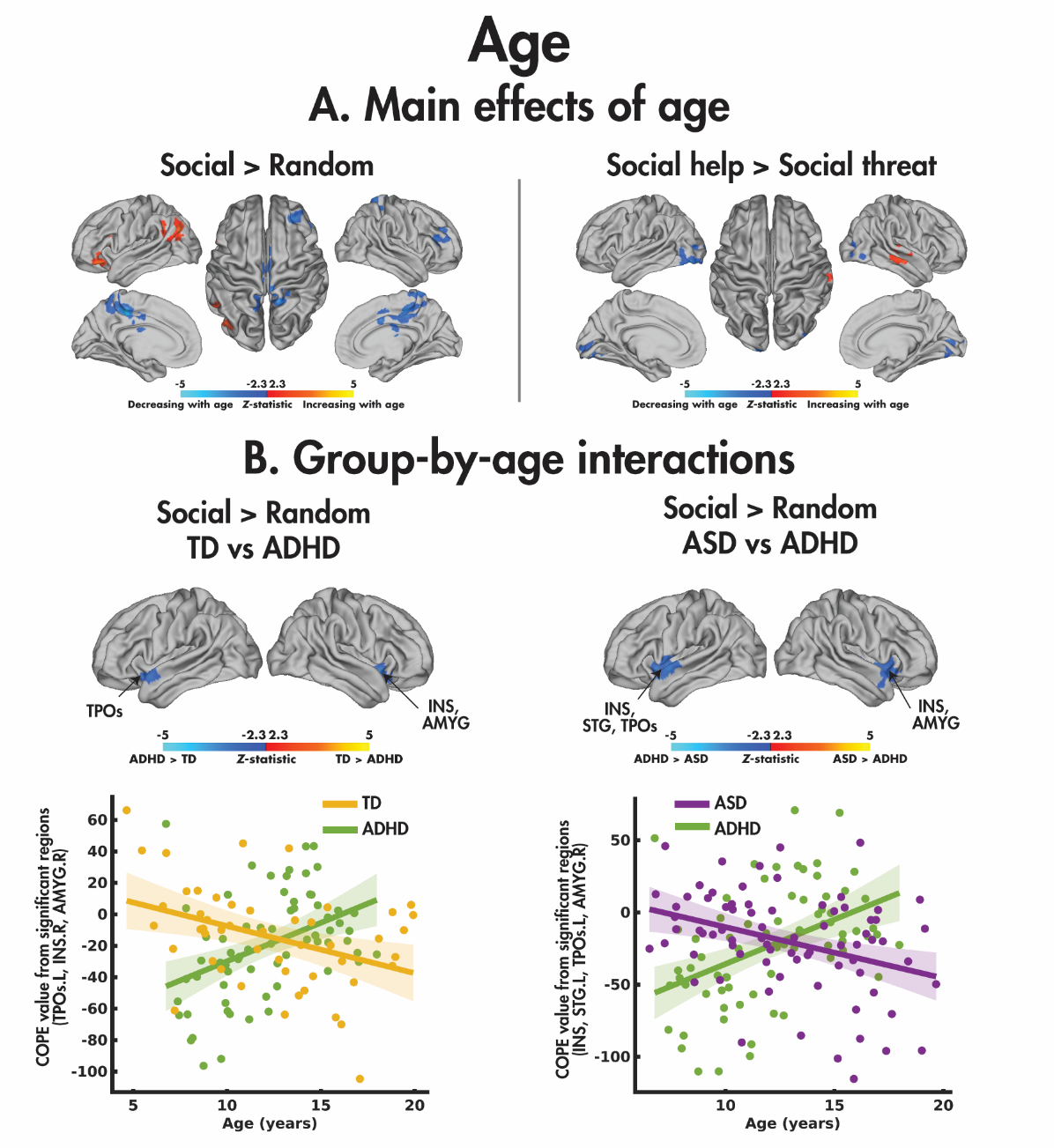


**Supplemental Figure 1**: Examining the effects of age in the SAT. Significant (*p*_corr_ < 0.05) main effects of age (A) for the social greater than random and social help greater than social threat contrasts are presented, and significant (*p*_corr_ < 0.05) diagnosis-by-age interactions (B) are shown for the social greater than random contrast between the TD, ASD and ADHD participants; the mean COPE values across significant regions were extracted and plotted with age to determine the directionality of the effect. No interactions were observed for the social help versus threat contrast.

Diagnosis-by-age interactions were found in the social greater than random contrast (**Supplemental Table 6**; **Supplemental Figure 1B**) in two clusters spanning the bilateral insulae. When examining the mean COPE values, compared to both the TD and ASD participants, the ADHD youth showed increasing activation with age in two clusters encompassing the bilateral insulae, engaging the insulae more to the random videos when young and shifting to the opposite pattern later in development. In contrast, TD and ASD participants showed age-related decreases, with little activation differential between the social and random videos when young and engaging the insulae more to random videos when older.

**Supplemental Table 6:** Brain regions showing significant diagnosis-by-age interaction for the social greater than random (*Z* > 2.3, *p*_corr_ < 0.05); no interactions were significant in the social help vs. threat conditions.

| **Contrast** | **Cluster** | **N_voxels_** | ***p*_corr_** | **Max**  ***Z*** | **Max Z coordinates**  **(x, y, z) (mm)** | **AAL regions** |
| --- | --- | --- | --- | --- | --- | --- |
| Slope ADHD  >  Slope TD | 1 | 903 | 5.45e^-4^ | 4.06 | (8, 8, -6) | INS.R, OLF.R, AMYG.R, CAU.R |
|  | 2 | 636 | 6.33e^-3^ | 4.30 | (-26, 4, -12) | OLF.L, TPOs.L, AMYG.L, PUT.L, INS.L |
| Slope ADHD  >  Slope ASD | 1 | 1113 | 9.35e^-5^ | 4.10 | (30, 4, -12) | INS.R, AMYG.R, PUT.R, ORBi.R |
|  | 2 | 915 | 4.91e^-4^ | 4.12 | (-38, 8, -22) | INS.L, STG.L, TPOs.L, ROL.L |

N_voxels_: Number of voxels, *p*_corr_: corrected *p*-value, *Z*: *Z*-statistic, AAL: Automated Anatomical Labeling atlas, INS: insula, OLF: olfactory cortex, AMYG; amygdala, CAU: caudate, TPOs: pole of the superior temporal gyrus, PUT: putamen, ORBi: orbital part of the inferior frontal gyrus, STG: superior temporal gyrus, ROL: rolandic operculum

**Brain-behaviour relations with data-driven subgroups**

Brain-behaviour relations were also investigated between the four observations (pairwise social vs random and social help vs threat) used in the data-driven subgrouping and the NEPSY-TM; results are presented in **Supplemental Table 5**.

**Supplemental Table 7:** Statistical results for the brain-behaviour relations ANCOVAs between the NEPSY-TM and the observations used in the data-driven subgrouping.

| **Observation** | **Behaviour**  **(*F*, *p*-value)** | **Dx-Behaviour interaction**  **(*F*, *p*-value)** |
| --- | --- | --- |
| Social > random | (1.44, 0.23) | (2.91, 0.09) |
| Random > social | (0.98, 0.32) | (4.80, 0.03) |
| Social help > social threat | (0.98, 0.32) | (2.00, 0.16) |
| Social threat > social help | (0.18, 0.67) | (2.95, 0.09) |

AAL: Automated Anatomical Labeling atlas, F: *F*-statistic, Dx: diagnosis, TD: typically developing, ASD: autism spectrum disorder, ADHD: attention-deficit/hyperactivity disorder

**Further examination of ASD and ADHD overlap**

After examining the pairwise contrasts, few differences emerged between the ASD and ADHD participants: only a cluster in the right fusiform gyrus was significant when contrasting the social help and social threat conditions. However, there were clusters that emerged as significant in the TD contrasts with one NDD group that were not present in the other NDD group (e.g., for the social versus random contrast, a occipital cluster was significant between TD and ADHD participants, but not TD and ASD participants). To establish why these clusters were not identified in the between NDD comparison, we replicated the rain cloud plots from **Figures 2** and **3**, this time including the diagnostic group omitted from the pairwise comparison (**Supplemental Figure 2**), and summarized the mean COPE values for each group (**Supplemental Table 8**). In all cases, the omitted group had a mean COPE value that fell between the two examined groups (e.g., for the occipital cluster, which was significantly different between TD and ADHD participants, the mean COPE value for the ASD participants was less than the TD children but greater than the ADHD children). The overlap between the TD, ASD and ADHD suggests that there is a continuum from typical to atypical ToM neural processing, and the order of this continuum is dependent on region of the brain. This supports taking a trans-diagnostic approach to studying these populations.


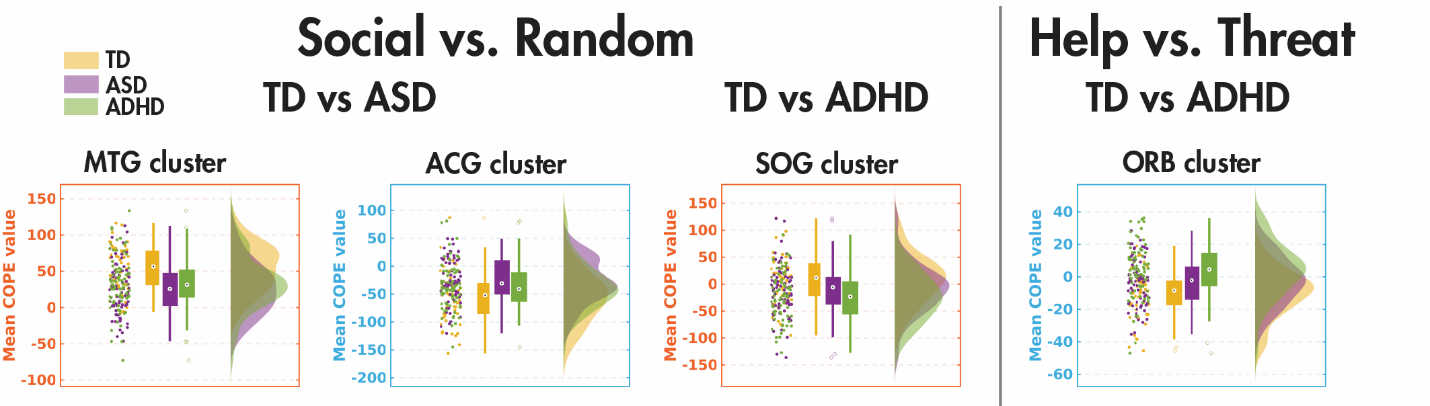


**Supplemental Figure 2:** Distribution of mean COPE values in all three groups for regions significantly different between TDs and one NDD group but not the other.

**Supplemental Table 8:** Means and standard deviations the COPE values for the clusters in **Supplemental Figure 2**.

|  | **MTG cluster** | **ACG cluster** | **SOG cluster** | **ORB cluster** |
| --- | --- | --- | --- | --- |
| TD | 54 ± 31 | -56 ± 47 | 9 ± 45 | -10 ± 15 |
| ASD | 25 ± 35 | -27 ± 39 | -10 ± 47 | -3 ± 14 |
| ADHD | 33 ± 38 | -37 ± 41 | -24 ± 44 | 4 ± 8 |

**References**

1 Klin A. Attributing social meaning to ambiguous visual stimuli in higher-functioning Autism and Asperger syndrome: The social attribution task. *J Child Psychol Psychiatry Allied Discip* 2000; **41**: 831–846.

2 Schultz RT, Grelotti DJ, Klin A, Kleinman J, Van Der Gaag C, Marois R *et al.* The role of the fusiform face area in social cognition: Implications for the pathobiology of autism. *Philos Trans R Soc B Biol Sci* 2003; **358**: 415–427.

3 *Diagnostic and statistical manual of mental disorders (DSM-IV-TR)*. American Psychiatric Publishing, 2000.

4 *Diagnostic and statistical manual of mental disorders (DSM-5®)*. American Psychiatric Publishing, 2013.

5 Wechsler D. *Wechsler Abbreviated Scales of Intelligence*. The Psychological Corporation: San Antonio, TX, 1999.

6 Wechsler D. *Weschler Intelligence Scale for Children*. 4th ed. The Psychological Corporation: San Antonio, TX, 2003.

7 Wechsler D. *Wechsler Intelligence Scale for Children*. 5th ed. Pearson: Bloomington, MN, 2014.

8 Wechsler D. *The Wechsler Preschool and Primary Scale of Intelligence*. 4th ed. The Psychological Corporation: San Antonio, TX, TX, 2012.

9 Achenbach TM, Rescorla LA. *Manual for the ASEBA school-age forms & profiles: An integrated system of multi-informant assessment*. Aseba: Burlington, VT, VT, 2001.

10 Berument SK, Rutter M, Lord C, Pickles A, Bailey A. Autism screening questionnaire: Diagnostic validity. *Br J Psychiatry* 1999; **175**: 444–451.

11 Harrison PL, Oakland T. *Adaptive behavior assessment system*. 2nd ed. The Psychological Corporation: San Antonio, TX, CA, 2003.

12 Korkman M, Kirk U, Kemp S. *NEPSY-II*. Pearson, 2007https://books.google.ca/books?id=UukMtwAACAAJ.

13 Smith SM. Fast robust automated brain extraction. *Hum Brain Mapp* 2002; **17**: 143–155.

14 Zhang Y, Brady M, Smith S. Segmentation of brain MR images through a hidden Markov random field model and the expectation-maximization algorithm. *IEEE Trans Med Imaging* 2001; **20**: 45–57.

15 Cox RW. AFNI: Software for analysis and visualization of functional magnetic resonance neuroimages. *Comput Biomed Res* 1996; **29**: 162–173.

16 Siegel JS, Power JD, Dubis JW, Vogel AC, Church JA, Schlaggar BL *et al.* Statistical improvements in functional magnetic resonance imaging analyses produced by censoring high-motion data points. *Hum Brain Mapp* 2014; **35**: 1981–1996.

17 Smith SM, Brady JM. SUSAN - A new approach to low level image processing. *Int J Comput Vis* 1997; **23**: 45–78.

18 Jenkinson M, Beckmann CF, Behrens TEJ, Woolrich MW, Smith SM. FSL. *Neuroimage* 2012; **62**: 782–790.

19 Salimi-Khorshidi G, Douaud G, Beckmann CF, Glasser MF, Griffanti L, Smith SM. Automatic denoising of functional MRI data: Combining independent component analysis and hierarchical fusion of classifiers. *Neuroimage* 2014; **90**: 449–468.

20 Griffanti L, Salimi-Khorshidi G, Beckmann CF, Auerbach EJ, Douaud G, Sexton CE *et al.* ICA-based artefact removal and accelerated fMRI acquisition for improved resting state network imaging. *Neuroimage* 2014. doi:10.1016/j.neuroimage.2014.03.034.

21 The Mathworks Inc. MATLAB. www.mathworks.com/products/matlab. 2016.

22 Strobl C, Boulesteix AL, Zeileis A, Hothorn T. Bias in random forest variable importance measures: Illustrations, sources and a solution. *BMC Bioinformatics* 2007; **8**. doi:10.1186/1471-2105-8-25.

23 Kaufman L, Rousseeuw PJ. *Finding groups in data: an introduction to cluster analysis*. John Wiley & Sons, 2009.

24 Luxburg U Von. A Tutorial on Spectral Clustering A Tutorial on Spectral Clustering. *Stat Comput* 2006; **17**: 395–416.
